# The effect of geometry and abduction angle on the stresses in cemented UHMWPE acetabular cups – finite element simulations and experimental tests

**DOI:** 10.1186/1475-925X-4-32

**Published:** 2005-05-17

**Authors:** Rami K Korhonen, Arto Koistinen, Yrjö T Konttinen, Seppo S Santavirta, Reijo Lappalainen

**Affiliations:** 1 Department of Applied Physics, University of Kuopio, P.O.Box 1627, FIN-70211 Kuopio, Finland; 2 Department of Medicine, Helsinki University Central Hospital, Biomedicum, P.O.Box 700, FIN-00029 Helsinki, Finland; ORTON Orthopaedic Hospital of the Invalid Foundation, FIN-00280 Helsinki, Finland; COXA Hospital for the Joint Replacement, FIN-33520 Tampere, Finland; 3 Department of Orthopaedics and Traumatology, University of Helsinki, P.O.Box 22, FIN-00014 Helsinki, Finland

## Abstract

**Background:**

Contact pressure of UHMWPE acetabular cup has been shown to correlate with wear in total hip replacement (THR). The aim of the present study was to test the hypotheses that the cup geometry, abduction angle, thickness and clearance can modify the stresses in cemented polyethylene cups.

**Methods:**

Acetabular cups with different geometries (Link^®^: IP and Lubinus eccentric) were tested cyclically in a simulator at 45° and 60° abduction angles. Finite element (FE) meshes were generated and two additional designs were reconstructed to test the effects of the cup clearance and thickness. Contact pressures at cup-head and cup-cement interfaces were calculated as a function of loading force at 45°, 60° and 80° abduction angles.

**Results:**

At the cup-head interface, IP experienced lower contact pressures than the Lubinus eccentric at low loading forces. However, at higher loading forces, much higher contact pressures were produced on the surface of IP cup. An increase in the abduction angle increased contact pressure in the IP model, but this did not occur to any major extent with the Lubinus eccentric model. At the cup-cement interface, IP experienced lower contact pressures. Increased clearance between cup and head increased contact pressure both at cup-head and cup-cement interfaces, whereas a decreased thickness of polyethylene layer increased contact pressure only at the cup-cement interface. FE results were consistent with experimental tests and acetabular cup deformations.

**Conclusion:**

FE analyses showed that geometrical design, thickness and abduction angle of the acetabular cup, as well as the clearance between the cup and head do change significantly the mechanical stresses experienced by a cemented UHMWPE acetabular cup. These factors should be taken into account in future development of THR prostheses. FE technique is a useful tool with which to address these issues.

## Background

Aseptic loosening is the most common cause for long-term failure of total hip replacement (THR). The success rate after revision surgery is much lower than that after the primary operation. Wear, which contributes to aseptic loosening [1-4], has been shown to correlate with the contact pressure of the UHMWPE acetabular cup [5,6]. An increase of the abduction angle of the acetabular cup has been shown to increase contact pressure and wear [7,8]. Different geometrical cup designs, polyethylene thicknesses and clearances between the cup and head have been demonstrated to modulate implant survival [2,7,9-15]. However, the combined effect of these parameters on polyethylene stress is still unclear. For instance, the different geometries of the acetabular cups may modify the contact pressure in different ways depending on the abduction angle. Parameters reducing contact pressure reduce plastic deformation and wear, and thus may diminish the risk for aseptic loosening.

Pre-clinical testing of hip implants prior to marketing is known to be important [16], since failure to perform proper testing may lead to unsatisfactory clinical results. Therefore, the geometries and orientations of acetabular cups should be optimized. Finite element (FE) modeling enables the evaluation of stress distribution throughout the THR prostheses [8,17] and an assessment of geometries and material properties which would be difficult or time-consuming to test experimentally.

In this study, two UHMWPE acetabular cups with different geometries and good long term clinical records, according to Scandinavian hip registers, were studied [18,19]. However, the cups belong to the same hip prosthesis system and their individual survivorships are not described separately in the registers. FE modeling and cyclic testing of hip implants in a simulator were used to test the hypotheses that the geometry, thickness and abduction angle of the cup, as well as the clearance between the femoral head and the cup could affect acetabular cup stress upon loading. Information from this study can be used for the future development of THR implants prior to their surgical installation.

## Methods

### Laboratory tests

Two UHMWPE acetabular cups* with different designs (Waldemar LINK GmbH & Co., Hamburg, Germany: IP, n = 4 and Lubinus eccentric (with a snap-fit primary locking mechanism), n = 8) were tested experimentally under a cyclic load [20]. Axial loading was used to investigate the plastic deformations occurring in the cups and to minimize wear.

**Figure 1:**
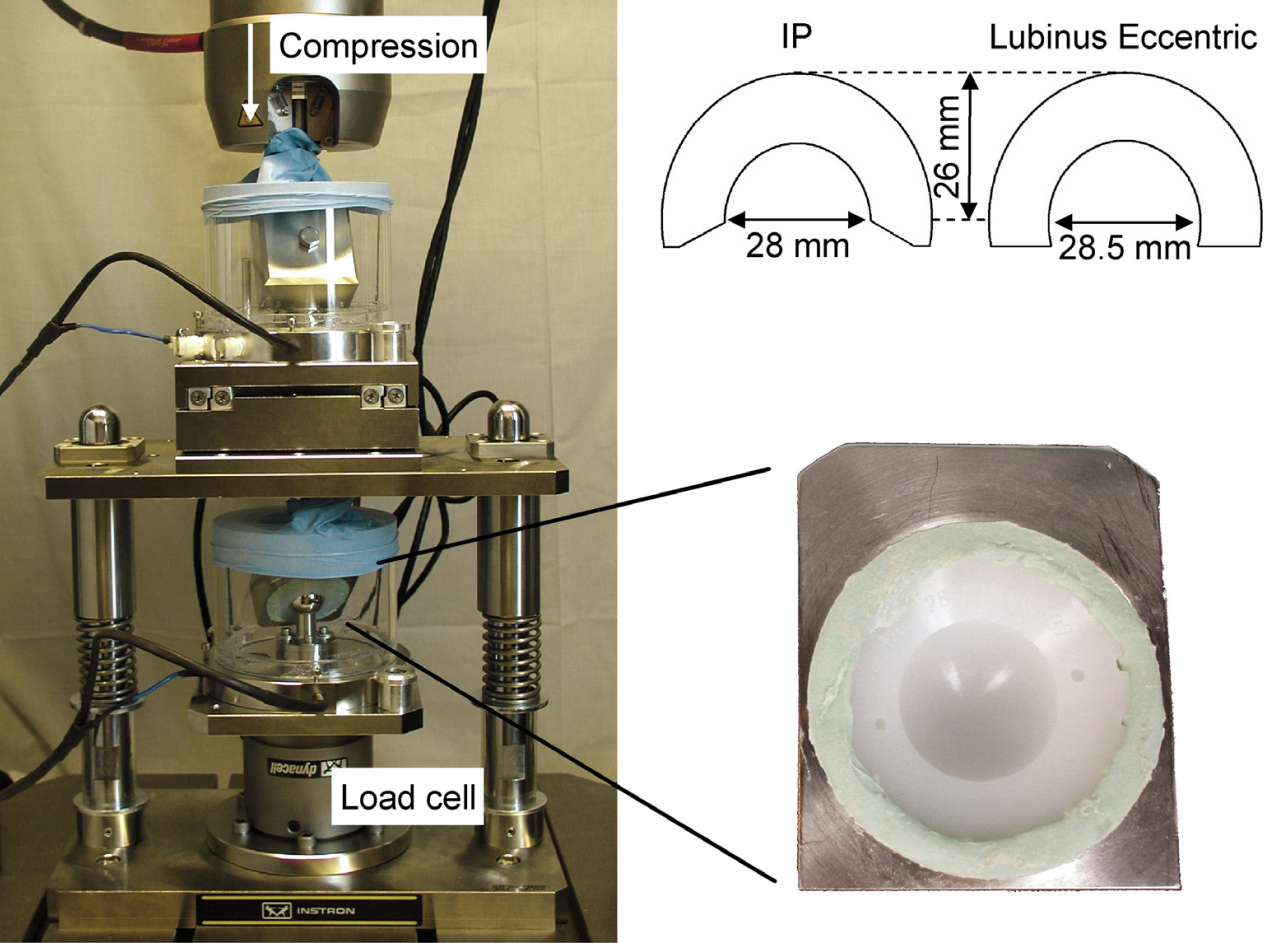
**Hip implant simulator and tested acetabular cups**. Two types of acetabular cups (up right, IP and Lubinus eccentric) were tested in a simulator (left) under cyclic axial loading. Two cups, fixed in a metal backing with bone cement, were tested at the same time.

Before the tests, the cups were fixed in stainless steel holders with bone cement (Palacos^® ^R-40 cum Gentamicin, Schering-Plough Europe, Brussels, Belgium). To speed up the experimental tests, two combinations of head, cup and cement were tested at the same time which led overall to six tests (Table 1, Fig. 1). Testings were carried out using a dynamic tester (Instron 8874, Instron Corporation, Canton, MA) at a 5 Hz frequency for 5 million cycles. The load profile was a Paul gait curve with a peak load of 3 kN, and abduction angles of 45° and 60° were used. Diluted bovine serum supplemented with EDTA and antibacterial agents was used as lubricant [21]. The serum was filtered through a 0.2 μm filter and had a total protein content of 25 mg/ml. Temperature (37°C) and pH (7) were regularly recorded.

**Table 1:** Summary of the parameters obtained from experimental tests and numerical simulations.

		Experimental tests		Finite element analyses			
			
		IP	Lubinus eccentric	IP1	Lubinus eccentric	IP2	Lubinus concentric
Compression (μm)	45°	Mean: 115 (n = 1)	Mean: 129 (n = 2)	98	132	-	-
			Range: 125–132				
	60°	Mean: 123 (n = 1)	Mean: 140 (n = 2)	107	133	-	-
			Range: 129–150				
							
Penetration rate (μm/million cycles)	45°	Mean: 11.8 (n = 2)	Mean: 9.5 (n = 4)	-	-	-	-
		Range: 11.6–11.9	Range: 8.9–10.2				
	60°	Mean: 14.7 (n = 2)	Mean: 11.2 (n = 4)	-	-	-	-
		Range: 14.2–15.2	Range: 10.0–11.9				
							
Von Mises stress	45°	-	-	Fig. 3	Fig. 3	Fig. 3	Fig. 3
	60°	-	-	"	"	"	"
							
Contact pressure and/or area	45°	Fig. 6	Fig. 6	Figs. 4-8	Figs. 4-8	Figs. 4-8	Figs. 4-8
	60°	-	-	"	"	"	"

45° and 60° refer to the abduction angle.

Experimental maximum compressions were analyzed from the initial loading cycles for two cup-cement combinations (12 acetabular cups, 6 experimental tests) at the same time (Fig. 1).

Penetration rates of the cups, *i.e*. plastic deformations, were calculated after 5 million loading cycles.

During the tests, compressions of whole measurement systems, *i.e*. compressions of femoral heads into the cup-cement combinations during each cycle, were recorded. Maximum compression values during the initial cycle were used for the validation of the finite element models. As the compression was applied according to Fig. 1, the final value for one cup-cement combination was approximated to be half of the recorded value, and consequently six compression values were obtained (Table 1).

After 5 million cycles, the penetration rates (μm / million cycles) of the acetabular cups, *i.e*. plastic deformations, were analyzed using a coordinate tester Dea Global C 091508 (Dea, Tourin, Italy). The penetration rates were evaluated as the displacement of the center of the spherical surface of the cups, which were mathematically estimated after the cyclic tests.

### Finite element modeling

**Figure 2:**
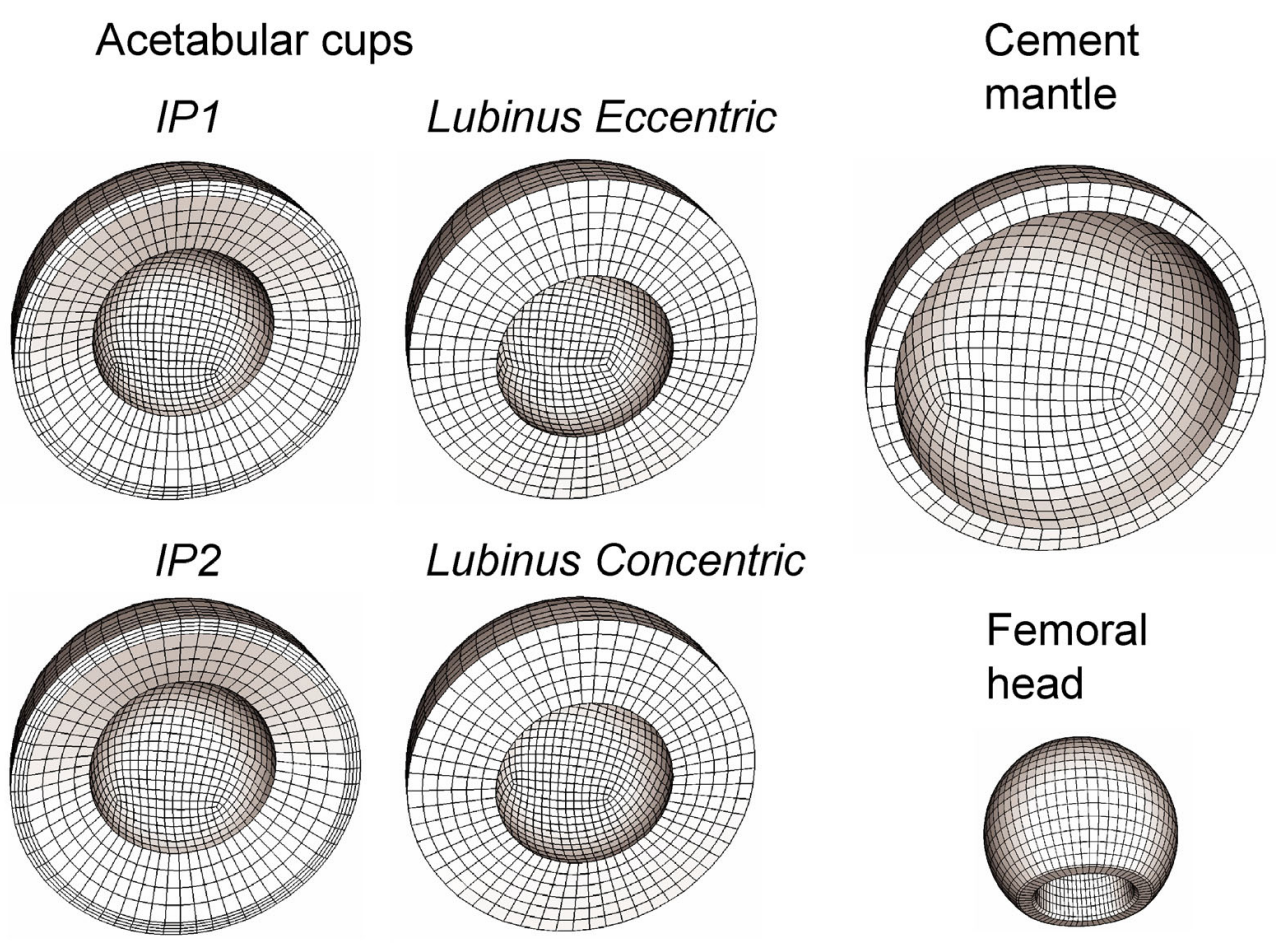
**Finite element meshes of the present study**. Four acetabular cups, cement mantle and femoral head were reconstructed by using hexahedral elastic elements.

The femoral head-acetabular cup-cement complexes, used in experimental tests, were processed for finite element analyses. In addition, two new designs were reconstructed to investigate the effect of clearance and polyethylene thickness on the contact pressure. Thus, altogether four 3-D meshes consisting of 15834 – 16072 hexahedral elements (Fig. 2) were created:

1) Lubinus eccentric (inner diameter of acetabular cup = 28.5 mm, experimentally tested),

2) Lubinus concentric, (inner diameter of acetabular cup = 28.5 mm, virtual design),

3) IP1 (inner diameter of acetabular cup = 28 mm, experimentally tested),

4) IP2 (inner diameter of acetabular cup = 28.5 mm, virtual design).

The outer diameter of all acetabular cups was 52 mm and the diameter of the femoral head was 28 mm. Lubinus concentric was reconstructed to analyse differences between eccentric and concentric cups and, consequently, the effect of polyethylene thickness. The effect of clearance was studied by increasing the inner diameter of the IP cup (IP1 → IP2) to correspond to the diameter of Lubinus eccentric/concentric. Young's moduli (*E*) for femoral head, acetabular cups and bone cement, as determined experimentally, were 193 GPa, 0.69 GPa and 2.74 GPa, respectively. A friction coefficient of 0.05 was assumed for the contact between acetabular cup and femoral head [22]. The cup-cement interface was fixed and the boundary constaint was applied for the outer surface of the cement layer to simulate the metal backing used in the experimental tests.

First, the compressions of the femoral heads into the acetabular cup – cement combinations were analyzed using 3 kN loading force and compared with corresponding experimental findings. Second, the effect of abduction angle on the stresses of acetabular cups was investigated with different loading forces in order to simulate different body weights performing regular daily activities and exceptionally high impact loads. In addition to the experimentally used abduction angles of 45° and 60°, simulations were also conducted using an angle of 80°. Von Mises stress distributions in the cups and contact pressures both at cup-head and cup-cement interfaces were studied. Abaqus code (v6.3, Hibbitt, Karlsson & Sorenssen, Inc., Pawtucket, RI, USA) was used for the FE simulations.

## Results

Experimentally, the maximum compressions of the femoral heads during the initial loading cycle using the IP and Lubinus eccentric acetabular cups were 115 μm and 129 μm, respectively, at the 45° abduction angle (Table 1). The corresponding values obtained in the FE simulations were 98 μm and 132 μm. At the abduction angle of 60°, experimental maximum compression values during the initial cycle using the IP and Lubinus eccentric cups were 123 μm and 140 μm, respectively. The corresponding values obtained in the FE simulations were 107 μm and 133 μm.

The measured penetration rates of the cups were 11.8 μm (11.6 – 11.9) and 9.5 μm (8.9 – 10.2) per million cycles for IP and Lubinus eccentric, respectively, at the abduction angle of 45° (Table 1). The corresponding values at the 60° angle, 14.7 μm (14.2 – 15.2) and 11.2 μm (10.0 – 11.9), were significantly higher (p < 0.05, Wilcoxon signed ranks test, n = 6 + 6). The penetration rates were significantly (p < 0.05, Mann-Whitney U-test, n = 4 + 8) higher for IP than for Lubinus eccentric.

**Figure 3:**
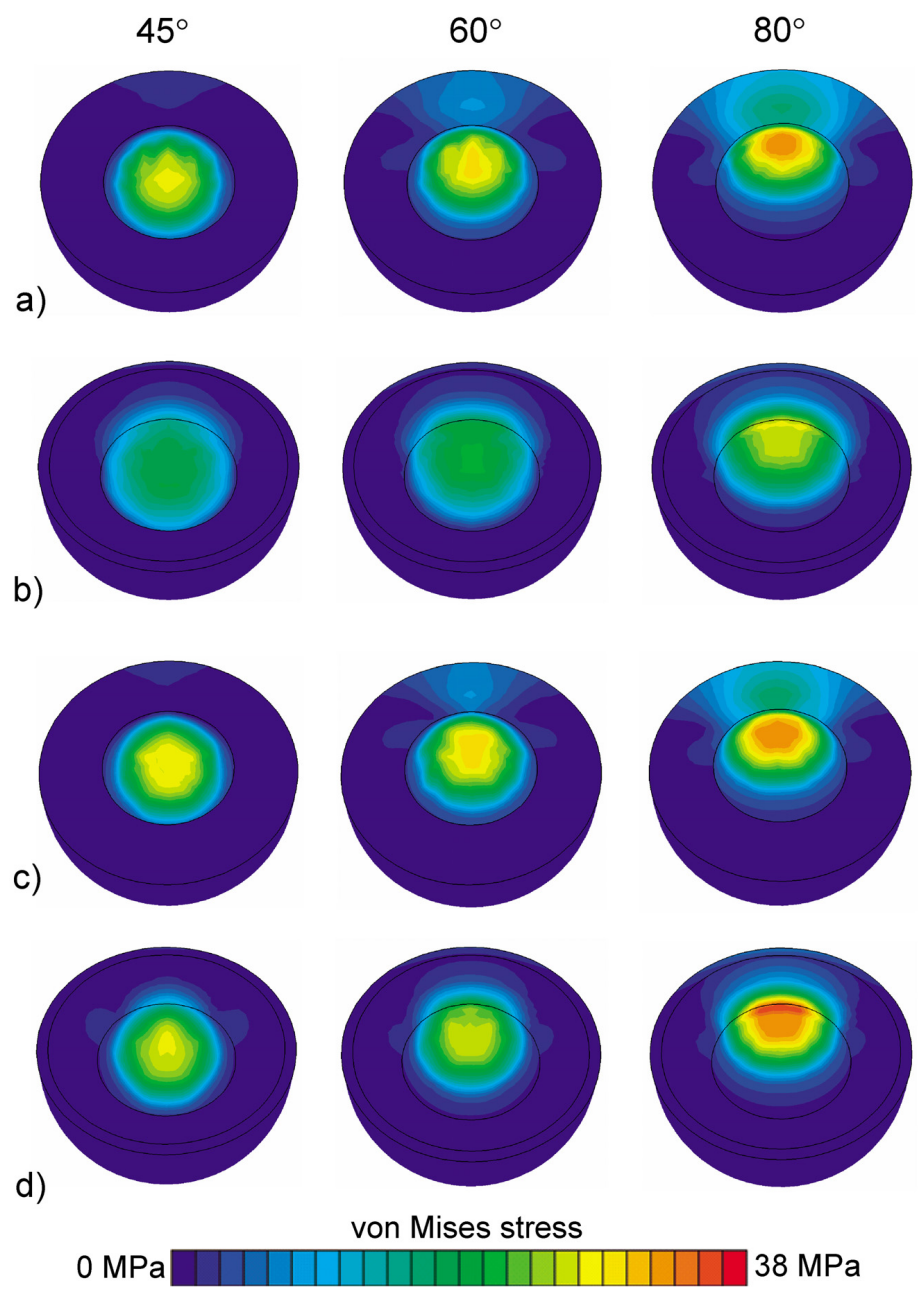
**Stress distribution in the acetabular cups**. Von Mises stresses of a) Lubinus eccentric, b) IP1, c) Lubinus concentric and d) IP2 models at 45°, 60° abduction angles.

FE simulations disclosed that there were different stress distributions (von Mises stress) in Lubinus eccentric and IP cups (Fig. 3). Due to the varying geometries, the direction of the stress was different. The decrease of the thickness of the Lubinus acetabular cup (eccentric *versus *concentric) did not affect the stress distribution, but the increase in the gap between the head and the IP cup had a major influence on the von Mises stresses.

**Figure 4:**
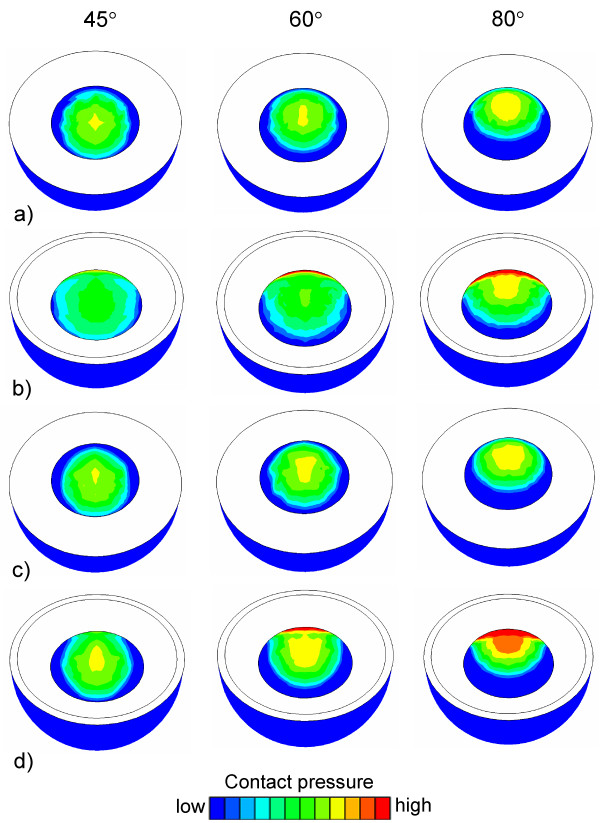
**Contact pressure at the inner surface of acetabular cups at 45° 60° and 80° abduction angles. **a) Lubinus eccentric, b) IP1, c) Lubinus concentric and d) IP2.

**Figure 5:**
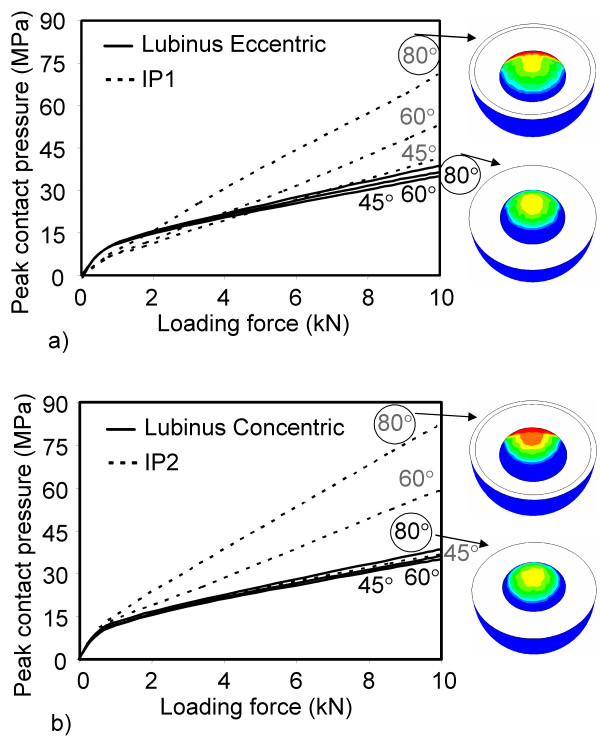
**Peak contact pressure at the cup-head interface as a function of loading force and abduction angle**. a) Lubinus eccentric and IP1, b) Lubinus concentric and IP2.

**Figure 6:**
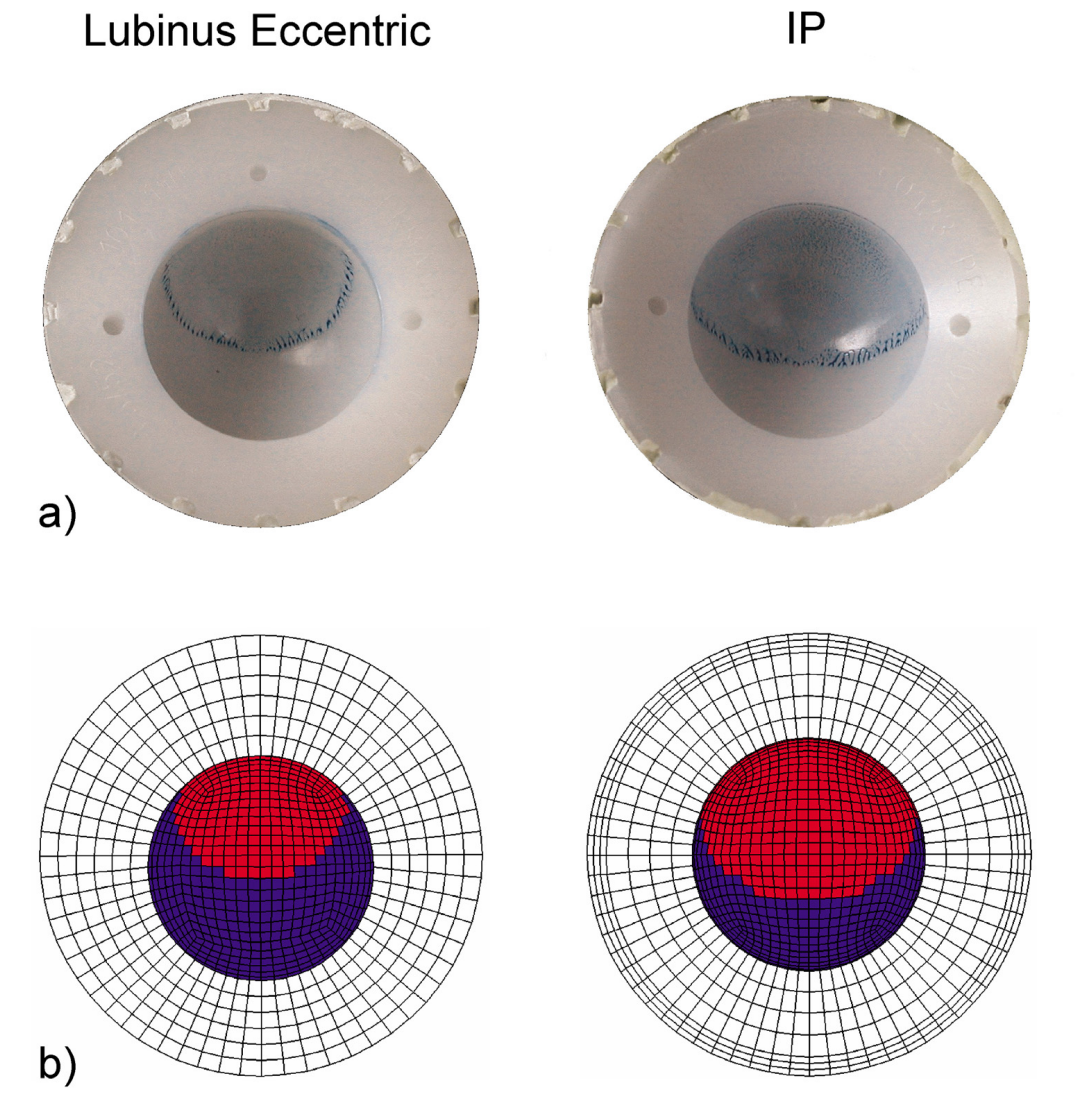
**Contact area between humeral head and acetabular cup**. Experimentally analyzed (a) and numerically simulated (b) contact area between Lubinus eccentric and IP acetabular cup and femoral head. After cyclic tests of hip implants in a simulator, the surfaces of UHMWPE cups were covered with a thin layer of diluted colourant (Tuschierpaste blau, Emil Alberts GmbH, Gevelsberg, Germany), and femoral heads were positioned in contact with the acetabular cups in the loading direction. After removing the femoral heads, the presence of blue color indicated the contact area. The contact area in the FE-simulations was analyzed under 3 kN axial load which was the maximum load in the experimental cyclic tests.

FE simulations showed that the abduction angle (45° – 80°) had only a minor influence on the contact pressure between the head and the Lubinus eccentric cup (Figs. 4a and 5a). In contrast, the contact pressure on the surface of IP cup increased notably as a function of the abduction angle (Figs. 4b and 5a). However, the larger contact area between the IP cup and head induced lower contact pressure at low loading forces on the surface of IP compared to the corresponding situation with Lubinus eccentric (Fig. 6). At high loads, the edge of the IP acetabular cup experienced very high contact pressures, especially at high abduction angles (60° and 80°). The difference in the contact pressure between the eccentric and concentric Lubinus models at the inner surface of the acetabular cup was not significant (Figs. 4a, c and 5). As the increase of the radius of the IP acetabular cup from 28 mm to 28.5 mm reduced the contact area, the peak contact pressure increased (Figs. 4b, d and 5).

**Figure 7:**
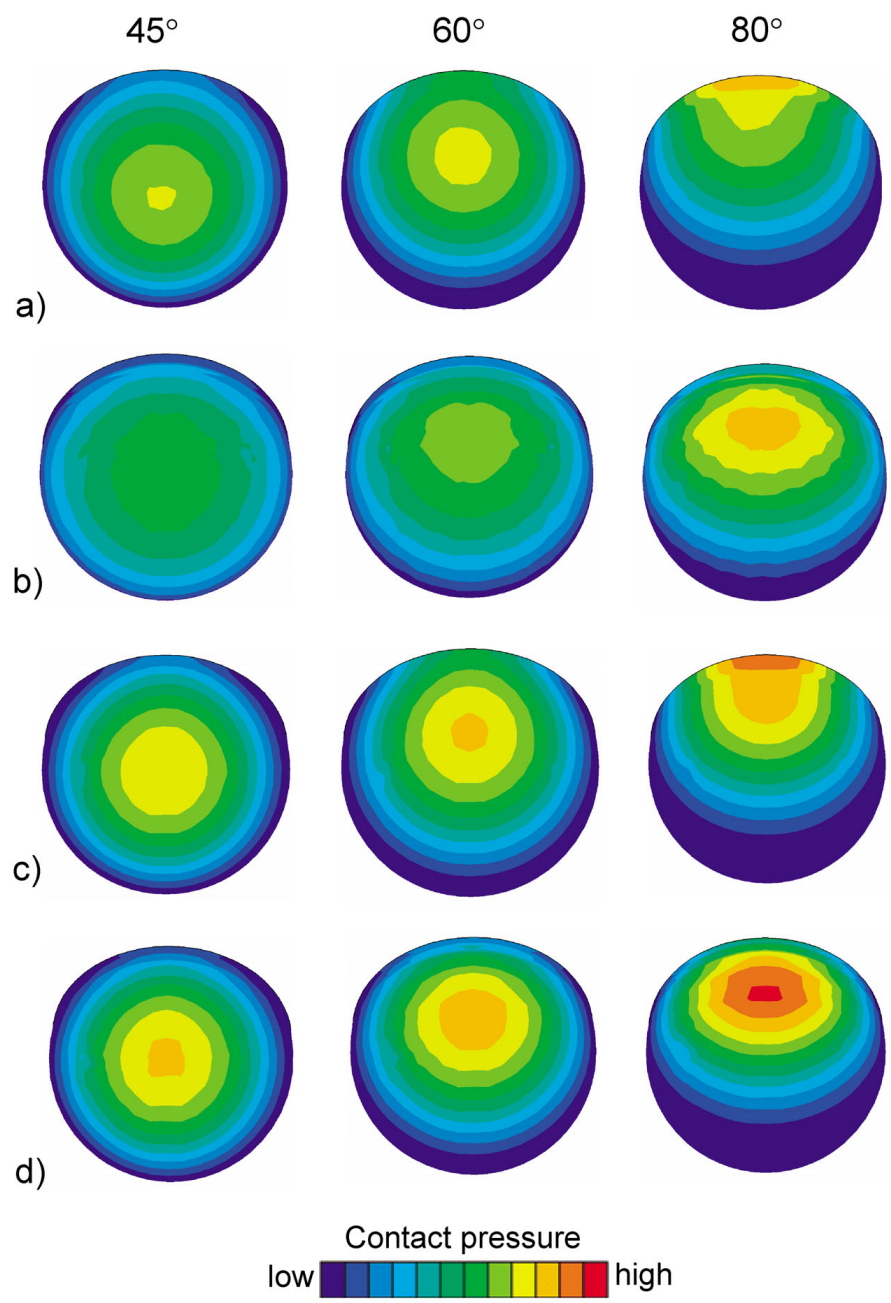
**Contact pressure at the outer surface of acetabular cups at 45°, 60° and 80° abduction angles**. a) Lubinus eccentric, b) IP1, c) Lubinus concentric and d) IP2

**Figure 8:**
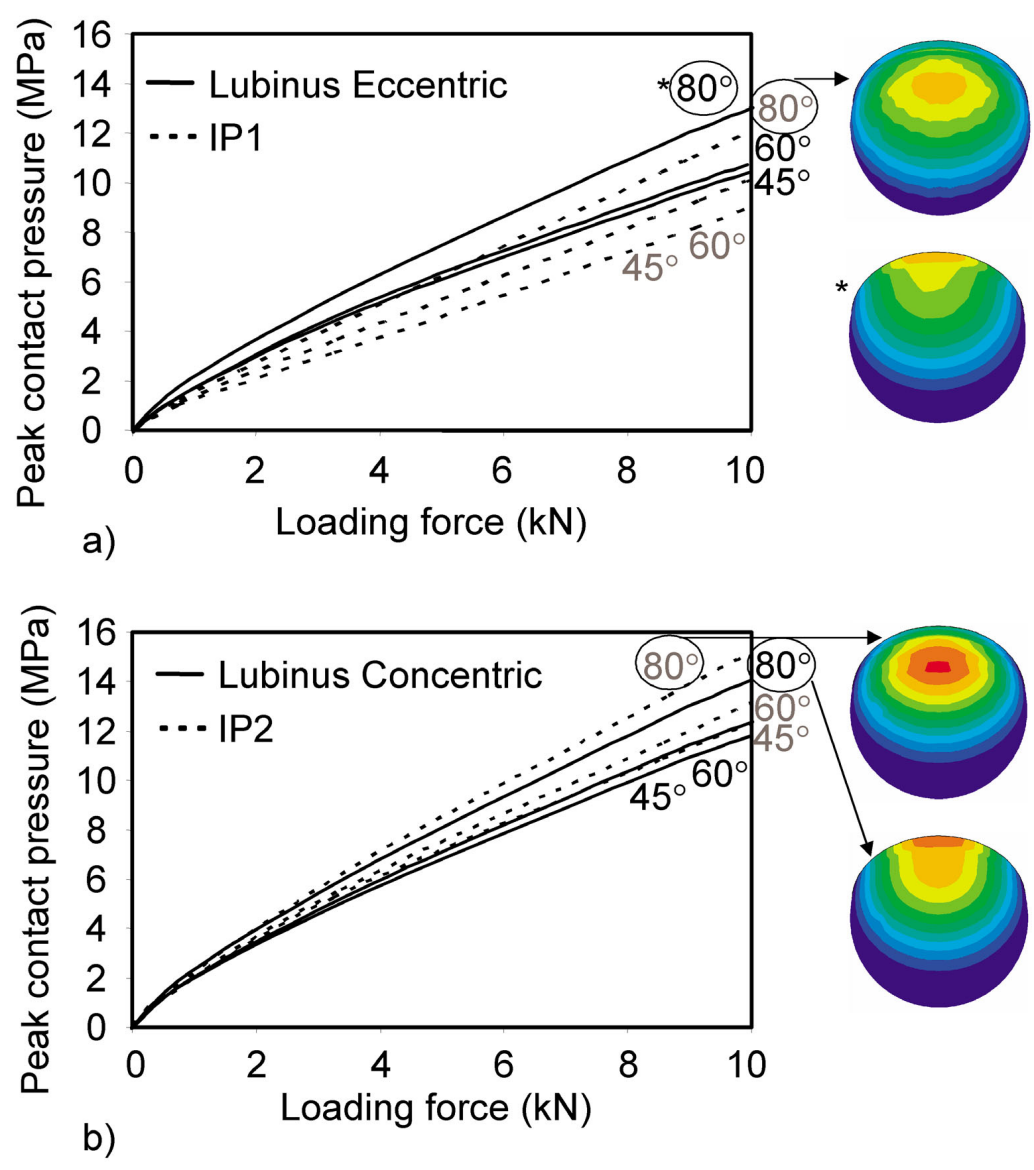
**Peak contact pressure at cup-cement interface as a function of loading force and abduction angle**. a) Lubinus eccentric and IP1, b) Lubinus concentric and IP2.

At the interface between the acetabular cup and the cement, the contact pressure increased as a function of abduction angle in all models (Figs. 7 and 8). Due to the larger contact area between cup and head and the smaller deformation of the cup, the contact pressure remained lower on the outer surface of the IP cup at all abduction angles (Figs. 7a, b and 8a). A decreased thickness of the acetabular cup (Lubinus eccentric → concentric) (Figs. 7a,c and 8) and the increased clearance between IP cup and femoral head (Figs. 7b,d and 8) elevated contact pressures on the outer surfaces of the cups.

## Discussion

In the present study, 3-D finite element analyses and experimental cyclic tests were used for the estimation of the combined effects of acetabular cup geometry and orientation on the stresses in cemented UHMWPE. It was found that IP and Lubinus eccentric acetabular cups, manufactured by Link^®^, were subjected to considerably different stress distributions and contact pressures both on the inner and outer surfaces of the cups. The abduction angle and thickness of the cup, as well as the clearance between the femoral head and the cup, contributed significantly to stress experienced by the acetabular cup.

Experimental and theoretical compressions during one loading cycle were consistent with each other. The slight discrepancies (2 – 15%) were probably due to the fact that the processing of UHMWPE induces uncertainties in the diameters of the cups, whereas in the finite element simulations, optimal diameters, supplied by the manufacturer, were used. On the other hand, the initial fit of the head-cup-cement complex may not have been perfect in all of the experimental configurations, and this may have induced higher compression values.

The experimentally determined penetration rates of the present study supported the conclusions based on the FE analyses. The experimental tests showed that the penetration rate increased significantly as a function of the abduction angle. This was consistent with the FE analyses which suggested that the contact pressure increased as a function of the abduction angle, even though this increase with the Lubinus model was relatively low. Even though the peak contact pressure remained lower on the inner surface of the IP acetabular cup compared to that on the Lubinus eccentric when simulating loading forces of less than 3 kN, the experimentally determined penetration rate was faster for the IP cup. The contact area between the IP cup and the femoral head was larger than that between Lubinus eccentric and the head (Fig. 6), which probably induced this discrepancy. The minor clearance and consequently high contact area have been shown to increase also volumetric wear [10,15]. Previously, in simulator tests, wear rates of Lubinus eccentric cups were lower than those of IP cups and some other models [23]. This advantageous effect might be due to the formation of a protective protein layer on the cup surface as a result of the more closed lubrication environment in the Lubinus eccentric design.

The behavior of contact pressure and penetration rate of the present study were consistent with earlier studies [8,9,12]. Jin *et al*. (1999) investigated contact pressure and area in eight combinations of femoral head and UHMWPE cup, and found similar behavior of contact pressure as a function of load as observed in the present study [9], although their model was 2-D axisymmetric and was not affected by the edge of the cup. Patil *et al*. (2003) studied the effect of abduction and anteversion angle of acetabular cup on the contact stress and wear of UHMWPE [8]. FE simulations of their study showed that peak contact stresses increased as a function of abduction angle, as was also found in the present study. Wear rates after testing in the hip simulator and linear head penetrations of the clinical study [8] as well as plastic deformations of the cups in the present study supported those findings. Some studies have suggested that the cup orientation plays minor role on the polyethylene wear, but more important is to determine the shape of the wear distribution [10,24]. In the present study, the distribution of von Mises stress and contact pressure were highly influenced by the cup geometry. In general, slight differences between the previous studies and the present study are probably due to differences in the acetabular cup geometries, material properties and the loading protocols used in the experimental tests and analytical or numerical analyses [8,25,26].

Abduction angle clearly differentiated the acetabular cups. Lubinus eccentric seemed to be relatively forgiving in dealing with the variations in the abduction angle, whereas at 60° and 80° abduction angles, the IP cup experienced high peak contact pressures, especially at high loading forces. Evidently, geometrical factors were behind this kind of behaviour. The snap-fit primary locking mechanism in Lubinus cup eliminated the effect of the abduction angle. In this model the contact area between the ball and cup did not change as a function of the abduction angle. This minimizes the variations in the contact pressure and indicates that the survival of Lubinus acetabular cup may not be so operation sensitive. On the other hand, even though the geometry of IP cup permits a large range of motion, the contact area between IP cup and head changed as a function of the abduction angle, modifying the contact pressure. Furthermore, the edge of the IP cup was the most critical area in elevating the contact pressure, pointing to an increased risk for the cup failure [27].

The thickness of the acetabular cup and the clearance between cup and femoral head may have significant clinical relevance [9-12,14,15]. For instance, in the present study, a 13% decrease of the thickness at the upper part of the acetabular cup (Lubinus eccentric *versus *concentric) increased the peak contact pressure at the cup-cement interface at a 45° abduction angle by 20% (3 kN load). Consequently, eccentricity may decrease the risk for cup rotation. The increase of the clearance between IP acetabular cup and femoral head from 0 to 0.25 mm elevated peak contact pressure at cup-head interface by 27% and at the cup-cement interface by 65% (3 kN load). Also earlier studies have shown that the increased clearance and decreased thickness raise peak contact pressure [9,12]. These percentual values suggest that rather minor changes in the geometrical parameters of UHMWPE acetabular cups may have significant consequences (e.g. rotation, wear, loosening) in long-term use.

In the present study, all of the materials and boundaries were kept similar to permit a comparative evaluation of acetabular cups with different geometrical designs. In the clinical setting, however, the bone support and joint capsule will affect the stresses to which the acetabular cups are subjected [28]. Also, the contact area between the ball and the cup increases during the so-called running-in period, which typically lasts less than a year [29]. The bearing surface of the polyethylene accommodates to the head radius through deformation, wear and creep, so that the effective clearance approaches zero. Therefore, the contact pressure and fracture risk decrease substantially. After the first year, the penetration rate of the head into the cup is fairly constant [29,30]. In long-term clinical use, this phenomenon may modify the behavior of the acetabular cups tested in the present study. Finally, cement fixation was assumed to extend up to the edge of the acetabular cups. However, in the clinical situation, the upper edge of the cup is not always fully supported by the cement mantle, especially at low abduction angles (<45°), inducing variations in the stress and wear concentrations of the cups.

A previous finite element model with elasto-plastic polyethylene cup proposed a perfect plastic yield after 8 MPa von Mises stress [15], which is lower than the maximum von Mises stress observed in the present study. However, the radiation-crosslinked UHMWPE tested in this study has a yield strength of 20 MPa or more (ASTM F648) [31] and the von Mises stress of IP cup at the abduction angles of 45° and 60° did not exceed 20 MPa under any load. In this study the cup was assumed to be elastic to enable analysis of potential peak stresses also at very high impact loads, not only during normal walking. Under low loads (< 3 MPa), the von Mises stress did not exceed 20 MPa in any cup design. In addition, if von Mises stresses are calculated for the IP acetabular cup at the 3 kN load, which was the maximum value used in the experimental tests of the present study, only approximately 8 MPa peak von Mises stress is reached at an abduction angle of 45°. Plasticity, nonlinear elasticity or viscoelasticity [8] of UHMWPE would have changed absolute pressure values, but not the conclusions of the present study.

FE modeling provides a rapid and inexpensive estimate of implant-related factors (e.g. geometry of the acetabular cup) and surgery-related factors (e.g. abduction angle of the implant), but the final judgement is based on laboratory tests and clinical data. The hip arthroplasty registers [18,19], used to obtain clinical reference data, do not differentiate between Lubinus eccentric and IP acetabular cups, but the present study points to differences in the performances of these cups. The results of this study indicate that it would be beneficial if cups of different designs were recorded separately in the registers, rather than combining cups from a single hip system. All experimental and numerical data presented in this study address the importance of optimizing the geometry and orientation of the acetabular cup before and during the operation, respectively. These are factors which may affect significantly failure of THR.

## Conclusion

FE analyses of the present study showed that the geometrical design, thickness and abduction angle of acetabular cup, as well as the clearance between cup and head modify the mechanical stresses experienced by UHMWPE. These factors should be taken into account in the future development of THR and the FE technique is a powerful tool for this purpose.

## Authors' contributions

RKK was involved in the design of the study, developed the FE models and calculated numerical predictions, as well as drafted the manuscript. AK participated in the design of the study, experimental tests and data analyses, as well as in the statistical analyses. YTK participated in the design of the study and coordination as well as helped in the manuscript preparation. SSS was involved in conceiving the study, coordination and manuscript preparation. RL participated in conceiving the study, coordination, experimental tests, FE model development and manuscript preparation. All authors have read and approved the final manuscript.

## Note

*Partially cross-linked polyethylene cups are processed according to ISO 5834-II and ASTM F648 standards. Cups are compression molded and gamma irradiated in a vacuum to a level of 27 kGy.
